# Disparities in veterinary education: a survey comparing first-generation and continuing-generation students in Germany

**DOI:** 10.3389/fvets.2025.1595643

**Published:** 2025-06-25

**Authors:** Alexandra Gloria Kracht, Marcus Georg Doherr, Katharina Charlotte Jensen

**Affiliations:** Freie Universität Berlin, School of Veterinary Medicine, Institute for Veterinary Epidemiology and Biostatistics, Berlin, Germany

**Keywords:** first generation students, equal opportunities, educational background, veterinary studies, Arbeiterkind, working class

## Abstract

**Introduction:**

Although university education in Germany is without tuition fees, the parental educational background influences the decision to study as well as the choice of subjects, and is linked to challenges that students face during the course of study. However, little is known about these aspects in veterinary medicine. In this study, the differences between the first-generation students (FGS) and continuing-generation students (CGS) in veterinary medicine, as well as the challenges (study entrance, financial situation, concerns about the future), are examined in order to identify any need for supportive action.

**Methods:**

The results of this study are based on a survey that was open to all German veterinary students in Spring 2023.

**Results:**

Responses from 1,525 students were analyzed (response rate 24%). A considerable proportion of veterinary students (40%) in Germany are FGS. They are more likely to rate their financial situation as poor (20%) or very poor (7%) than CGS (poor: 10%, very poor: 3%). Even though FGS and CGS work for income alongside their studies in similar proportions (FGS: 71%; CGS: 67%), the motivation was different: CGS are more likely to work in order to earn extra money (CGS: 68%; FGS: 49%) and FGS because other sources of income are insufficient (FGS: 34%; CGS: 12%). A similar proportion of FGS and CGS frequently or consistently considers dropping out of university (FGS: 10%; CGS: 11%). However, in FSG this is more often due to financial reasons (FGS: 28%, CGS: 16%). Almost all students have a high school-based university entrance qualification (97%). Final school grades of CGS were better on average than those of FGS. More FGS than CGS had already completed vocational training prior to their studies (FGS: 47%, CGS: 30%).

**Discussion:**

In conclusion, FGS and CGS differ in their own educational background and the financial challenges in veterinary education. It is therefore important to create an awareness of challenges related to the social background of veterinary students. Moreover, support services to counteract challenges of FGS (and CGS) are needed. There is a need for further research concerning the associations between parental educational background and academic and professional success in veterinary medicine.

## 1 Introduction

Providing equal opportunities is an important goal of many education systems—also in Germany (1, 2). Equal opportunities are intended to create social justice on the one hand (1) and promote efficiency and diversity in the world of work on the other (3). An important aspect of equal opportunities is the promotion of educational advancement. It has been shown that social background is still a decisive factor in whether someone starts a degree program (4–6). Students are traditionally categorized according to their social background: First-generation students (FGS) or working-class students (in German: Arbeiterkinder) are students where both parents do not have an academic educational background. If at least one parent has an academic educational background, the person belongs to the category of continuing-generation students [CGS; German: Akademikerkinder; (7)].

First-generation students are more likely to come from lower-income households (8) and, compared to CGS, are more likely to struggle with financial difficulties (6) and imposter feelings during their studies (9). FGS are less likely to be encouraged by their parents to study (8, 10, 11). In addition, FGS are more often inadequately prepared for their studies (12). Due to the lack of experience from their immediate environment, FGS can quickly become overwhelmed by the abundance of information about studying (7, 13, 14). FGS are also more likely to work alongside their studies (4) and have the feeling of being ‘different’ (15): they are caught between two worlds—the academic world and the world of origin. Thereby, the feeling of belonging can be challenging for FGS (14).

At the same time, however, positive characteristics are also attributed to FGS: personal qualities often found in FGS are innovative thinking and perseverance (16). Also, resilience is more pronounced among FGS (14, 17). A further argument in favor of supporting FGS is that they have often completed vocational training before studying (4) and therefore often already have the knowledge, skills and corresponding attitude for their later work, as well as for their studies. In Germany, vocational training usually last 3 years and combines school-based training with on-hand (practical) work.

As in other countries, there is currently a shortage of veterinarians in Germany (18, 19). This not only threatens the health of pets and livestock, but can also negatively impact food safety and human health in the sense of One Health (18, 19). While, on the one hand, research and discussions are taking place on how to prevent veterinarians from leaving the profession (19), there are also discussions about changing the university entrance qualification (20). In principle, the aim must be to admit and retain people who are committed to and enjoy their work as veterinarian. However, these considerations should also include the idea of equal opportunities. Social justice and inclusion should also be considered when admitting students to veterinary medicine programs (21), especially as FGS are underrepresented in the fields of medicine and dentistry (22)—and maybe also in veterinary medicine.

In Germany, there are various options for entering a university-based course degree program, with the best-known route being to obtain a qualifying high school degree (“Abitur” after 12 or 13 years of school). The term ‘non-traditional students’ refers to those who have not acquired their university entrance qualification through a school qualification (4). A rather rarely used route in Germany is entry to a university through a vocational training and working experience. Here it does not matter what school-leaving qualification the person has, which means that it is also possible to study at a university with a secondary school leaving certificate (after 10 years of school). However, the final high school grade (FHSG) plays a decisive role in admission to the veterinary medicine degree program: 30% of places are centrally allocated on the basis of final grades. The majority of study places (60%) are allocated via the university’s selection procedure, in which the final grade also accounts for a large part of the weighting. Only 10% of study places are allocated independently of high school grades. To date, little is known whether there are differences between FGS and CGS in terms of university entrance qualification.

In Germany, veterinary medicine can be studied at five Federal State funded universities (Freie Universität Berlin, University of Veterinary Medicine Hannover, Justus-Liebig University Giessen, University of Leipzig, Ludwig-Maximilians Universität München). In contrast to the Bologna-based Bachelor/Master system, the veterinary medicine curriculum is highly structured and concludes with a state examination after 5 ½ years (11 semesters). It is one of the degree programs with the highest weekly working hours (23). For instance, the weekly study-related working time at the Freie Universität Berlin is 41.4 h (24). The degree program offers little flexibility, as only a few courses can be freely chosen in terms of subject and time orientation. In addition, the standard period of study of 11 semesters is longer than most other degree programs (23). In other degree programs ending in state examination, such as law, pharmacy and medicine, the proportion of FGS is lower than in other courses of study (22). In human medicine and dentistry, for example, 74 per cent of students in Germany are CGS (4). Comparable data are not yet available for veterinary medicine.

In Germany, only administrative costs are charged by the universities, which amount to a few hundred euros per semester. Nevertheless, students have to provide for their living expenses. They are financed either by their parents, supported by the government of Germany stipend system (“BAföG”) or scholarships, take out a loan or work alongside their studies. So far, information on how veterinary students finance their living expenses in Germany is not available. There is also no data on mandatory health insurance contributions by students; until the age of 25 they are typically included in the family insurance of the parents, but afterwards have to arrange and pay for their own coverage.

Against this background—the endeavor for equal opportunities, the shortage of specialists in veterinary medicine and the many hurdles involved in studying veterinary medicine—we conducted a survey among German veterinary students in order to answer the following questions:

1. What is the proportion of FGS in veterinary students?
2. How does the previous educational background of FGS differ from that of CGS in veterinary medicine?
3. Do FGS more frequently struggle with financial problems than CGS in veterinary medicine? If so, what are these particular challenges and barriers?
4. Are there differences between FGS and CGS with regard to their future planning? Do FGS think more often about dropping out of university and less often to write a doctoral thesis after graduation than CGS?

The overall objective is to raise awareness that veterinary students differ in their (educational) background, and to identify options for action to provide equal opportunities for FGS when compared to CGS.

## 2 Materials and methods

### 2.1 Conceptualization of the questionnaire

After an in-depth literature review, the research questions were specified and a set of hypotheses was drawn up. Based on established questionnaires such as the German social survey [“Sozialerhebung”; (4, 22)], a first draft of the questionnaire was created to address these hypotheses. The questionnaire was divided into five sections: 1. demographics, employment and health insurance, 2. entry into studies and previous educational biography, 3. studies, 4. social background, 5. personality. In particular, the last two blocks were based on the Social Survey (4, 22) in order to ensure comparability. The questions created were entered into the online survey system LimeSurvey© (version 5.x) and subjected to a pre-test by the authors, doctoral students at the institute and 17 veterinary students representing the target population. Based on the comments and remarks, the questionnaire was finalized.

### 2.2 The survey

The study was approved by the Central Ethics Committee of the Freie Universität Berlin (No.: ZEA24039). Before completing the survey, the students were informed about the topic and the processing of the data. The data protection statements had to be confirmed by the participants.

The students were contacted by email via the General Students’ Committee of the five German universities or lecturers. They were able to take part in the survey via an open online link. The invitation containing the link was sent out at least two, at some faculties three times. In addition, the survey was advertised through social networks and WhatsApp groups. The survey was open from March 17 to May 7, 2023.

### 2.3 The questionnaire

The questionnaire is available at an online and as Supplementary material repository^1^ in the original language (German); it contained 58 questions. The majority of the questions were single-choice, often ordinal (Likert) scales. Some questions were multiple-choice options or short free text fields. Not all participants had to answer all questions due to filter questions. Completing the survey took around 15 min on average. Apart from the filter-based questions, all were mandatory, but the default answer for some Likert scales was “no response,” so participants did not have to answer it.

Section 1 (demographics, employment and health insurance) was comprised of 11 questions, mainly on demographic data. In addition to age, gender, marital status, childcare, data on finances, employment, the extent of employment, the reasons for employment, the influence of employment, and health insurance was gathered.

Section 2 contained 31 questions on the choice of study, admission and current study status. The place of study, start of studies and semester were asked at the beginning of the section. This was followed by questions on the highest school-leaving qualification, the date on which the qualification was obtained and the average grade. Students were also asked about lower school-leaving qualifications and previous vocational training and other previous courses of study.

In Section 3, participants were asked eight study-related questions assessing the perception and planning of participants. The first questions covered the self-assessment of the financial situation, as well as the organizational aspects in the last 12 months and during the practical year (PY). The PY is a period in veterinary studies during which students can choose internships in line with their specialist interests and specified standards. It comprises two semesters shortly before the end of their studies. Then, questions about organizational problems regarding changes to plans and last-minute appointments for exam preparation meetings were asked. This was followed by questions about thoughts of dropping out of the current veterinary degree course and, in the case of an affirmative answer option, the reasons for the thoughts of dropping out. The last question in this section included a question on the intention to write a doctoral thesis after finishing studies.

In Section 4, the educational background of their mother and father was obtained to classify the students into FGS and CGS. Although different relationship models are possible, e.g., that students grew up with two men as parents, we opted for the classic model in order to be able to compare these questions with comparable studies such as the German social survey.

Section 5 contained four questions adapted from the social survey on personality, as well as self-efficacy and a self-assessment of character. Analysis of the responses to these questions will be part of another publication. The survey ended with a free text field in which participants could ask questions or make suggestions.

### 2.4 Data processing and analyses

At the end of the survey period, responses were exported to Microsoft Excel (version 2019) and checked for plausibility. Incompletely answered questionnaires were excluded from the analysis, as the decisive question for this study regarding the parents’ educational qualifications was at the end of the survey. Clearly implausible answers (like being born in 200) were excluded by setting the information to missing. The age (in years) was calculated from the year of birth to the date of participation. Free text answers, such as the course of educational training, were sorted into corresponding categories. Where necessary, categories were combined. For example, part-time jobs with 80–100 h per month and jobs with 100 or more hours per month were combined into one category (>80 h/ month) to increase the respective frequencies. On the basis of their parents’ qualifications, the students were categorized as FGS or CGS. Descriptive analyses were carried out using the statistics program SPSS (version 27). Differences between FGS and CGS were determined using the chi-square (CS) or Fisher’s Exact Test (FET). Differences concerning quantitative variables (age, beginning of studying, semester, FHSG) were analyzed using Mann–Whitney-U-tests (MWU) as these variables were not normally distributed. The data was presented graphically using histograms and bar charts.

## 3 Results

### 3.1 Participants

**Figure 1:**
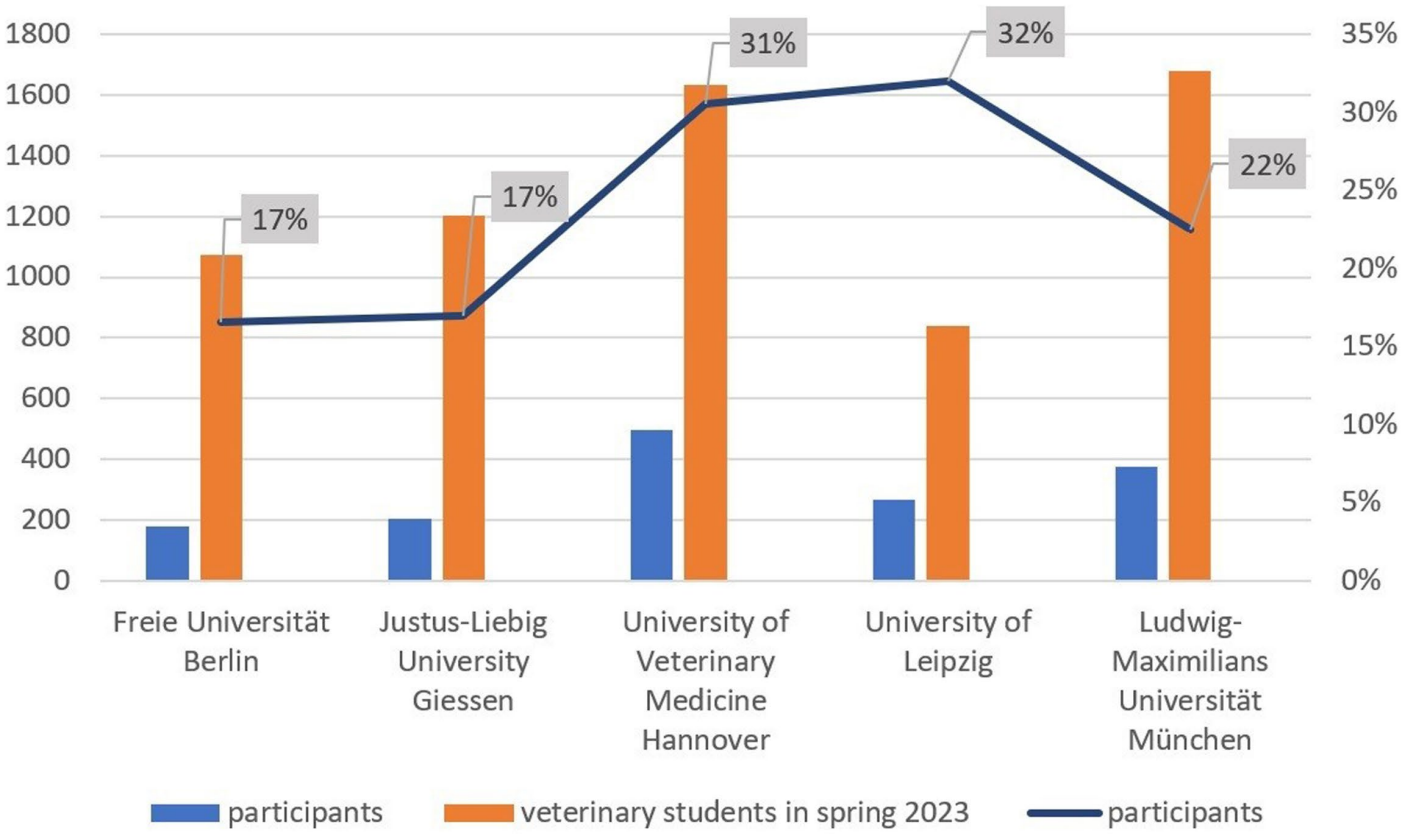
Proportion of participants (*n* = 1,525) in relation to the number of German veterinary students in 2023.

**Figure 2:**
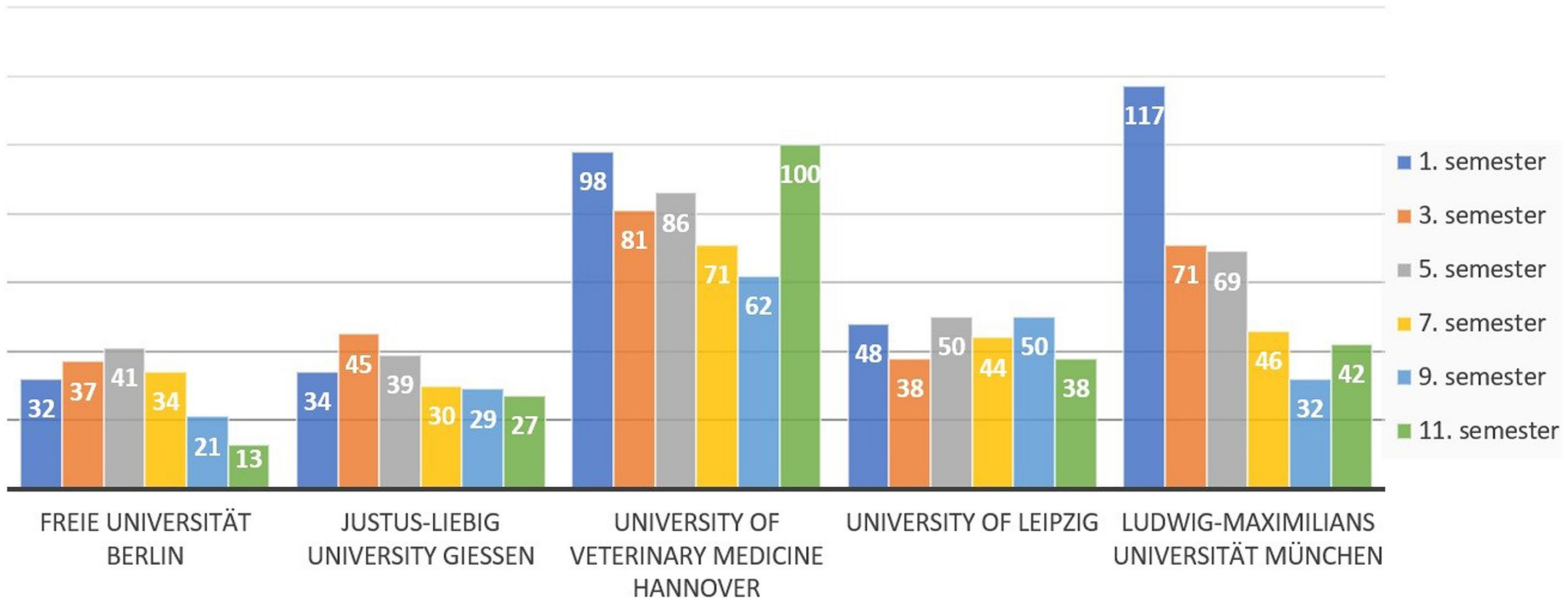
Distribution of participants (*n* = 1,525) in relation to semester and university in a survey of veterinary students in Germany.

A total of 2,078 people completed the survey. Of these, 553 did not complete the survey in full. Most people abandoned the survey on the first page (444 people). As the question about the parents’ education was only answered on the penultimate page and this question is of central importance for this manuscript, only the answers of individuals that fully completed the survey (*n* = 1,525) were analyzed. According to the German university enrolment data, 6,422 students were enrolled in veterinary medicine at the time of the survey. Therefore, 23.8% of veterinary students in Germany took part in the survey, varying between 17 and 33% between universities (Figure 1). The distribution of participants by academic semester across universities is shown in Figure 2. Overall, it can be seen that the distribution is reasonably balanced, although first semester students from Munich are slightly overrepresented.

In terms of gender distribution, there was a ratio of 92.1% female (*n* = 1,405) to 7.2% male (*n* = 110) veterinary medicine students, as well as 0.7% (*n* = 10) who could not or did not want to assign themselves to a gender. The respondents were on average 24.5 years old.

### 3.2 Proportions of FGS and CGS

Of the 1,525 participants, not all students stated their parents’ highest education degree, but 1,498 students could be divided into the two groups. Of the respondents, 60.1% (*n* = 901) stated that at least one parent had a university degree; these were classified as CGS. Correspondingly, 39.9% (*n* = 597) of participants were classified as FGS.

On average, FGS were older than CGS (mean: CGS: 24.0; FGS 25.1; MWU *p* < 0.001). With regard to the semester and also the year of entering veterinary school, there was no difference between FGS and CGS (CS: *p* = 0.628 and *p* = 0.475 respectively), so that the age difference is not due to a higher semester, but to a higher age when entering veterinary studies.

### 3.3 Admission process, school certificates & grades, and educational training

Continuing-generation students are almost twice as likely to be admitted to veterinary medicine via the best final high school grade (FHSG) quota (CGS 22.3%, *n* = 201; FGS 12.7%, *n* = 76). On the other hand, FGS are slightly more likely to be admitted to veterinary medicine via the university selection process and the additional aptitude quota, as shown in Table 1. In Germany, the FHSG is scaled from 0.7–4.0 with lower scores indicating a better result. If we look at the FHSG in separate categories of FGS (*n* = 571) and CGS (*n* = 881), the median FHSG for FGS was 1.90, whereas the median for CGS was 1.70 (MWU: *p* < 0.001), whereby not every person of the veterinary students has obtained an Abitur or has stated the grade.

**Table 1:** Admission process to veterinary studies of German students (*n* = 1,498).

	Total		CGS		FGS	
	*n*	%	*n*	%	*n*	%
Admission process						
Best high school grade	277	18.5	201	22.3	76	12.7
University selection procedure	784	51.4	453	50.3	322	53.9
Additional aptitude quota	213	14.2	112	12.4	101	16.9
Quota for international students	15	1.0	10	1.1	5	0.8
Hardship application	2	0.1	2	0.2	0	0
Second course of studies	31	2.1	21	2.3	10	1.7
Lottery procedure	66	4.4	40	4.4	26	4.4
No information^*^	119	7.9	62	6.9	57	9.5
Total	1,498	100	901	100	597	100

* This category includes students who started their studies abroad.

**Figure 3:**
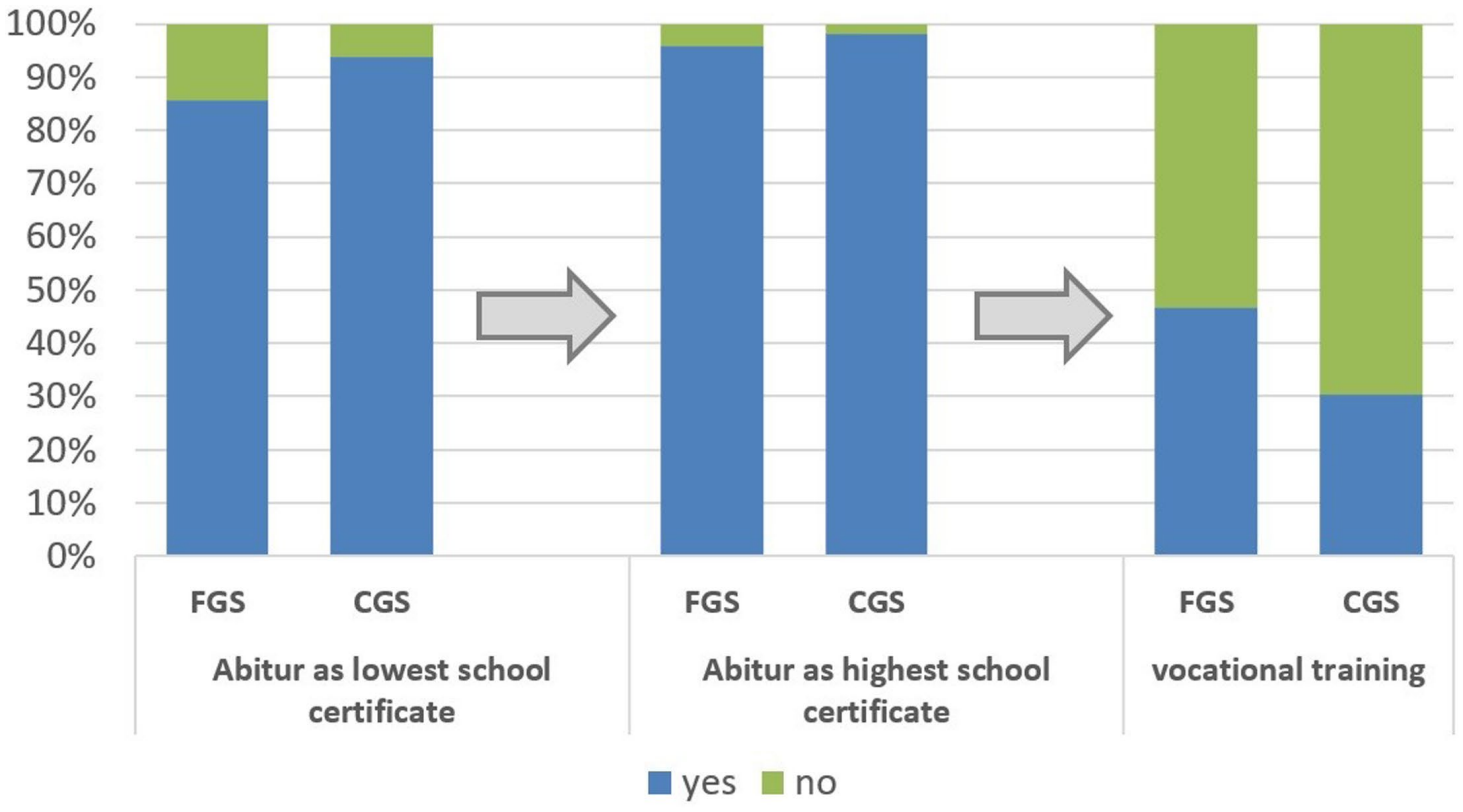
Comparison of educational biographies of German first- and continuing-generation students of veterinary medicine (*n* = 1,498); “Abitur” is the highest German school leaving certificate. Persons who obtained Abitur as lowest school certificate obtained this directly. Persons who have a lower school leaving school certificate than Abitur obtained first a secondary school leaving certificate or “Fachhochschulreife.”

When comparing the educational biographies of veterinary students separately for FGS and CGS, 846 CGS (93.9%) and 511 FGS (85.6%) stated that they had not obtained a lower (such as secondary) school leaving certificate before obtaining their high school certificate. Of the CGS, 273 (30.3%) had completed at least one vocational training. In contrast, 278 FGS (46.6%) had completed at least one vocational training (Figure 3).

Overall, 34.2% of the students were dual-qualified (*n* = 521), i.e., they had both a vocational and a school-leaving qualification. If this is broken down between the cohorts, there is a clear trend towards CGS having fewer dual qualifications than FGS (CGS: 28.4%, *n* = 256; FGS: 42.9%, *n* = 256; CS: *p* < 0.001).

### 3.4 Financial situation, employment, organization and future planning

**Figure 4:**
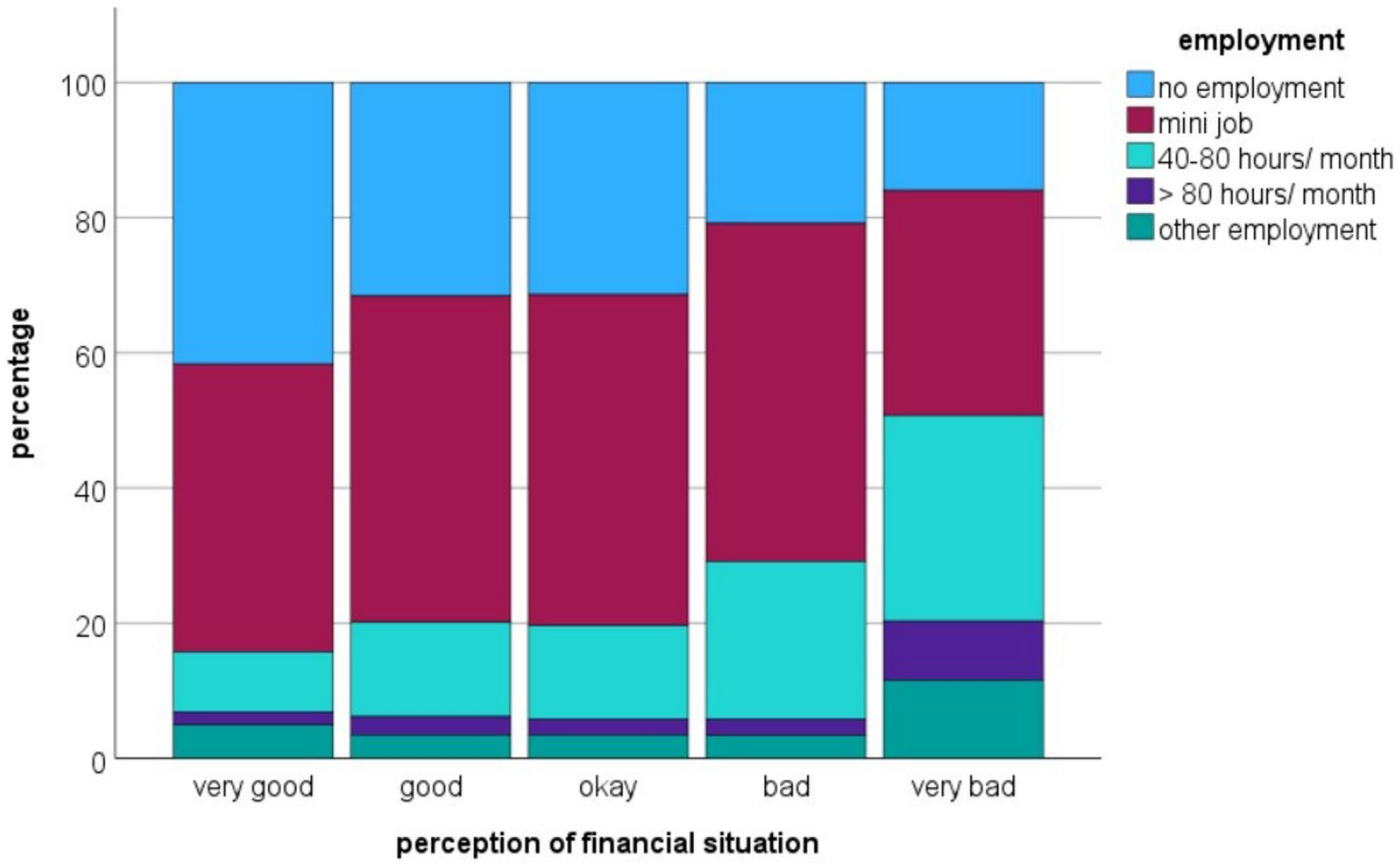
Association between the assessment of the financial situation and the employment in a survey of German veterinary students (*n* = 1,525); mini job: marginal employment, up to 43 h per month.

Looking at the subjective assessment of their financial situation, FGS perceived their situation significantly worse than CGS (CS: *p* < 0.001; Table 2). The subjective perception of the financial situation depending on employment is shown in Figure 4. Interestingly, people who rated their financial situation as “5—very bad” were significantly more likely to work 40 or more hours per month. This was observed in both cohorts.

**Table 2:** Perception of the financial situation of German veterinary students from a survey including 1,493 participants.

	Total		CGS		FGS	
	*n*	%	*n*	%	*n*	%
Financial situation						
1—very good	322	21.6	256	28.5	66	11.1
2	479	32.1	311	34.7	168	28.2
3	422	28.3	219	24.4	203	34.1
4	202	13.5	85	9.5	117	19.6
5—very bad	68	4.6	26	2.9	42	7.0
Total	1,493	100	897	100	596	100

Comparison between CGS and FGS (Chi-Square test): *p* < 0.0001.

Of the students surveyed, 31.6% (*n* = 473 of 1,498) stated that they were not employed. These were 298 CGS (33.1% of a total of 901) and 175 FGS (29.3% of 597). Of the remaining respondents, 46.9% (*n* = 703 of 1,498) were regularly employed—i.e. also during the running semester—whereby this affected slightly fewer CGS (*n* = 408; 45.3%) than FGS (*n* = 295; 49.4%). Only 5.9% (*n* = 89 of 1,498) were only employed during the lecture-free period and 15.6% (*n* = 233 of 1,498) were employed occasionally or when needed. This distribution did not differ significantly between FGS and CGS (FET: *p* = 0.073).

Among the employed students (964 participants), both FGS (72.3%, *n* = 287) and CGS (73.4%, *n* = 416) were predominantly employed in the context of a mini-job (marginal employment, up to 43 h per month depending on wage). In the category of students working 40–80 h per month, FGS were almost identically represented at 23.2% (*n* = 92) as CGS at 22.9% (*n* = 130). Significantly fewer students, 4.5% (*n* = 18) of FGS and 3.7% (*n* = 21) of CGS, worked 80 or more hours per month. This difference was not statistically significant (CS: *p* = 0.802).

**Figure 5:**
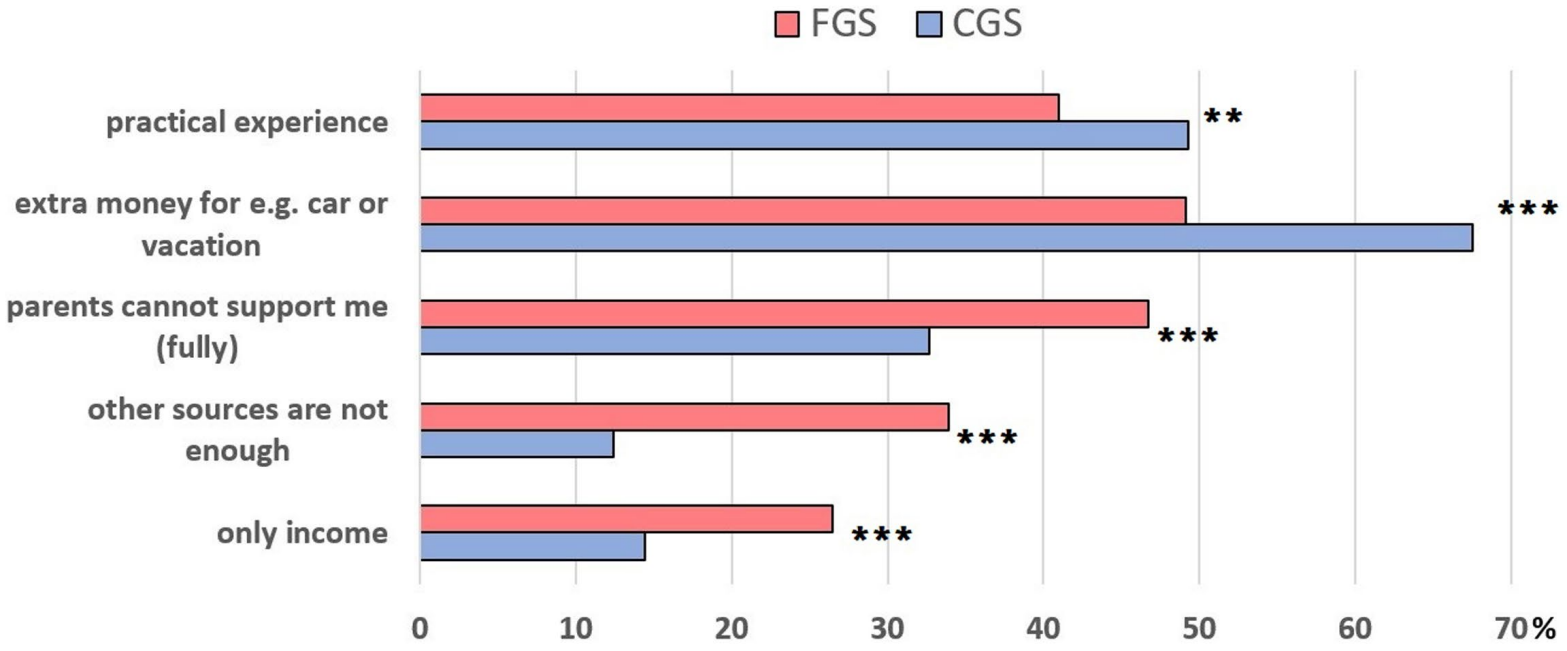
Reasons for employment from a survey of German veterinary students (*n* = 1,081) Comparison between FGS and CGS (Chi-Square test): ** *p* < 0.01; *** *p* < 0.001.

Students who stated that they were employed had the opportunity to provide reasons for their employment (Figure 5). Of those, 26.5% of FGS (*n* = 112) and 14.4% of CGS (*n* = 87) stated that their employment was their sole source of income (CS: *p* < 0.001). When assessing the other answer options, CGS were significantly more likely to state that they were employed due to practical experience or to be able to afford something special, while FGS were significantly more likely to state that they were employed because other sources were insufficient.

**Figure 6:**
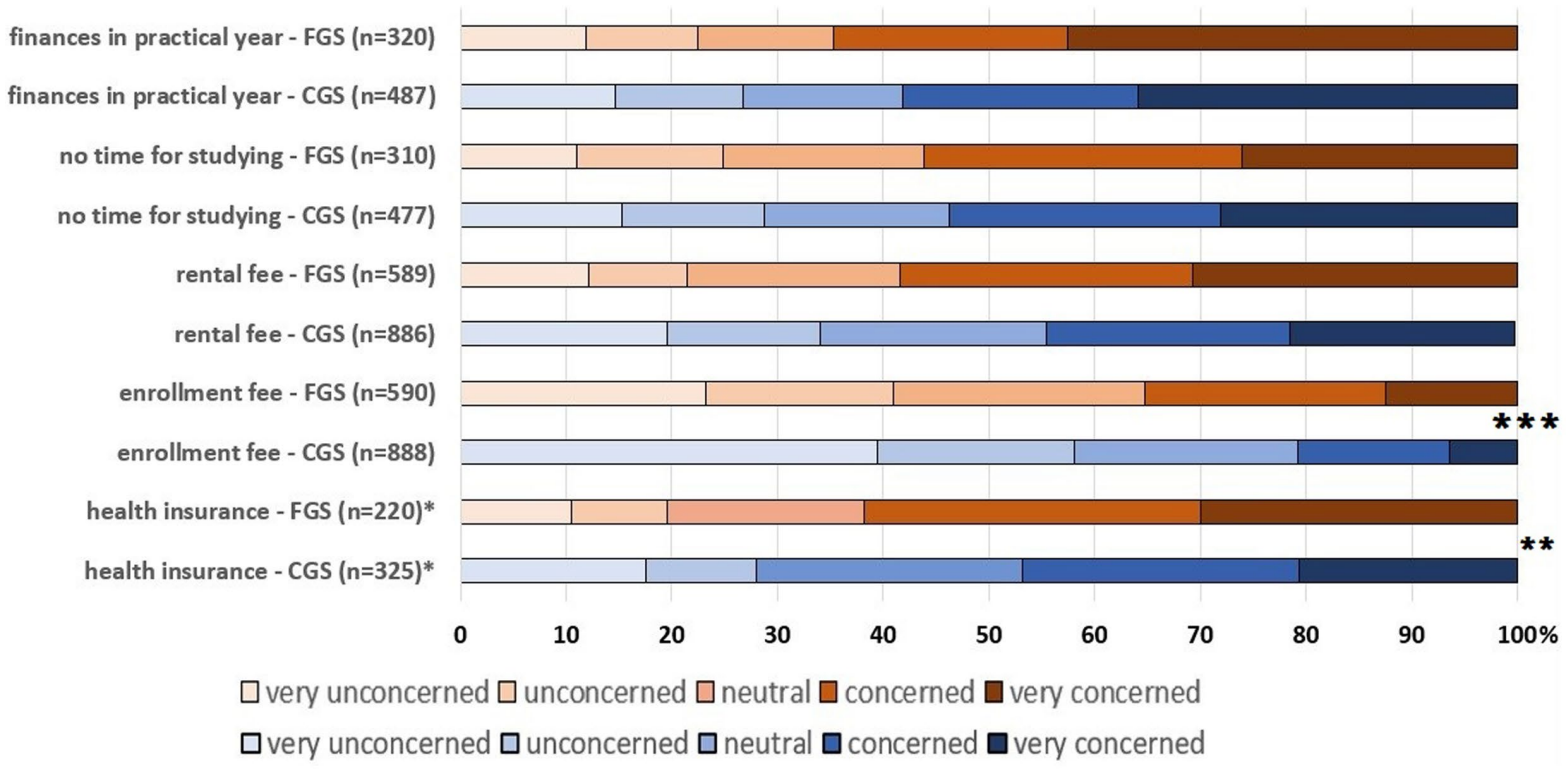
Assessment of concerns of German first- and continuing-generation students of veterinary medicine regarding financial and organizational aspects during their studies (*n* = 1,498). * this question was only answered by those students who paid the health insurance themselves Comparison between FGS and CGS (Chi-Square test): ** *p* < 0.01; *** *p* < 0.001.

When comparing participants’ responses to more differentiated aspects of the financial and organizational situation, FGS indicated a higher level of concern about the four financial aspects than CGS (Figure 6). The question about the costs of health insurance was only answered by those students who bore these costs themselves. This affected 39.5% (*n* = 354) of the CGS and 39.1% (*n* = 230) of the FGS. Thus, there was no difference between CGS and FGS who had to pay their contribution themselves, only the perceived burden from the subsequent filter question was more pronounced for FGS (*p*-value = 0.014). In contrast, there were no differences between FGS and CGS in the statement about organization (“time to learn”; CS: *p* = 0.349).

Students who had to work alongside their studies, have to look after children or have other commitments could experience organizational problems due to late course assignments, changes to plans or last-minute appointments (Table 3). There were no differences between CGS and FGS concerning this aspect (CS: *p* = 0.147). Those people who “sometimes/ often/ always” had problems with planning often stated that the rescheduling of employment was particularly stressful (56.5%), while caring for children (3.9%) and other relatives in need for care (9.5%) was less often mentioned as a challenge.

**Table 3:** Responses to the question “Students who work, care for children or have other commitments additional to their courses can get in trouble when courses or exams are planned in the short term.

	Total		CGS		FGS	
	*n*	%	*n*	%	*n*	%
I experienced changes but they do not affect me at all	422	28.2	260	28.9	162	27.1
I sometimes have problems	652	43.5	399	44.3	253	42.4
I often have problems	244	16.3	136	15.1	108	18.1
I always have problems	91	6.1	47	5.2	44	7.4
I never experienced a short-term change of plans	89	5.9	59	6.5	30	5.0
Total	1,498	100	901	100	597	100

How often did this problem affect you?” from a survey among German veterinary students (*n* = 1,498). Comparison between CGS and FGS (Chi-Square test): *p* = 0.147.

### 3.5 Future planning

#### 3.5.1 Thoughts of dropping out

Students’ responses related to the thoughts of dropping out indicate that around 11% of participants had thoughts of dropping out of the course of study (Table 4). There was no difference between the two groups (CS: *p* = 0.950).

**Table 4:** Responses to the question “Have you thought about quitting your veterinary studies in the last 12 months?” from a survey among German veterinary students (*n* = 1,485).

	Total		CGS		FGS	
	*n*	%	*n*	%	*n*	%
Never	915	61.6	551	61.6	364	61.6
Sometimes	415	27.9	248	27.7	167	28.3
Often	117	7.9	73	8.2	44	7.4
More or less the whole time	38	2.6	22	2.5	16	2.7
Total	1,485	100	894	100	591	100

Comparison between CGS and FGS (Chi-Square test): *p* = 0.950.

The participants who had thoughts of dropping out often stated that this was due to the study requirements [CGS: 63.8% (*n* = 60); FGS: 73.3% (*n* = 44)]. It was striking that financial reasons were more frequently a reason for thoughts of dropping out among FGS (CGS: 6.5%, *n* = 6 of 93; FGS: 20%, *n* = 12 of 60). Acute health problems were a reason for thoughts of dropping out for 51 of 151 respondents (33.8%). Chronic health problems (13.3%; 20 of 150) and pregnancy (5.3%; 8 of 150) were of minor importance for thoughts about dropping out of university. Concerning the last three mentioned reasons, no differences between FGS and CGS were apparent.

**Figure 7:**
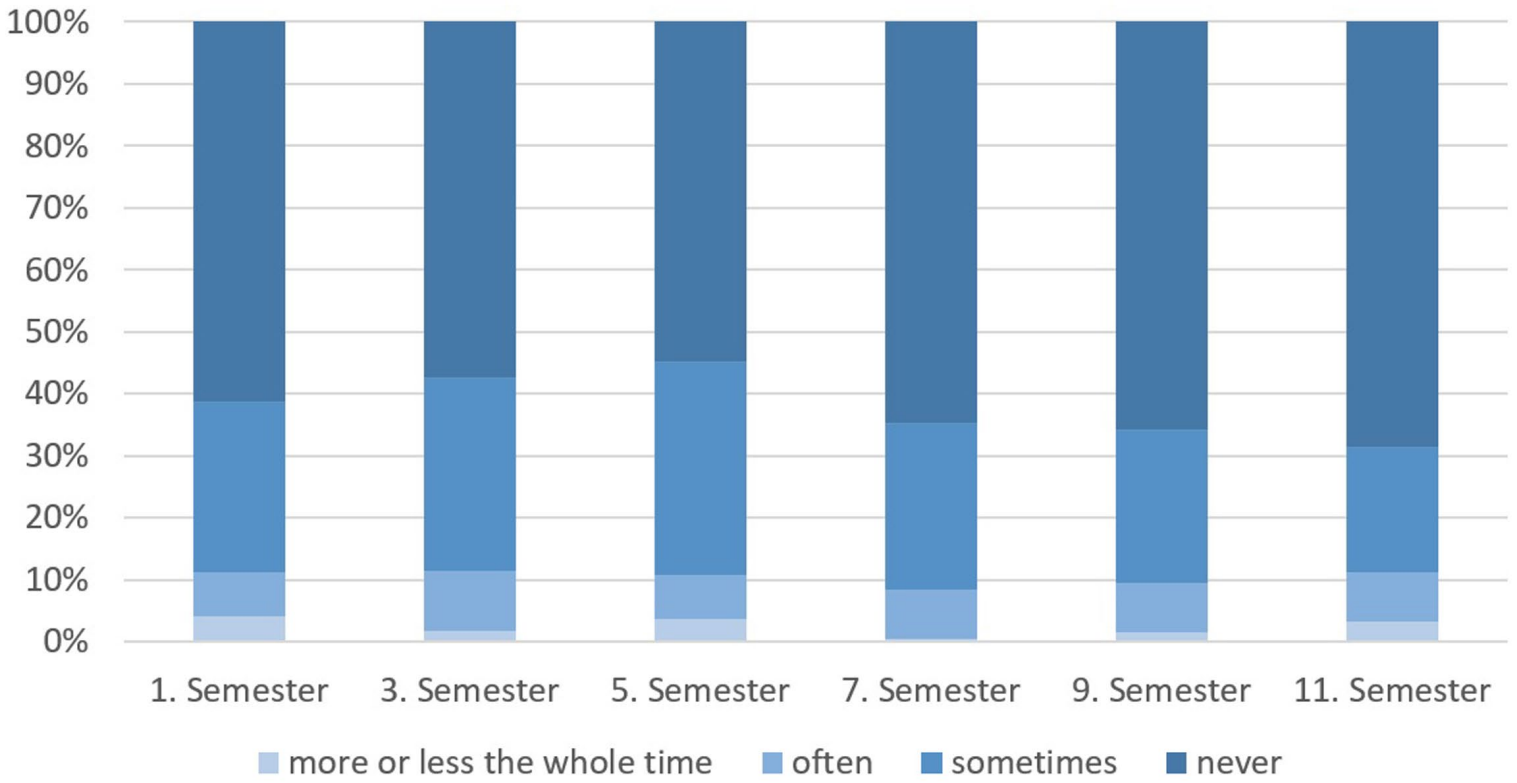
Thoughts of dropping out of veterinary studies during the different semesters of German veterinary students (*n* = 1,509).

**Figure 8:**
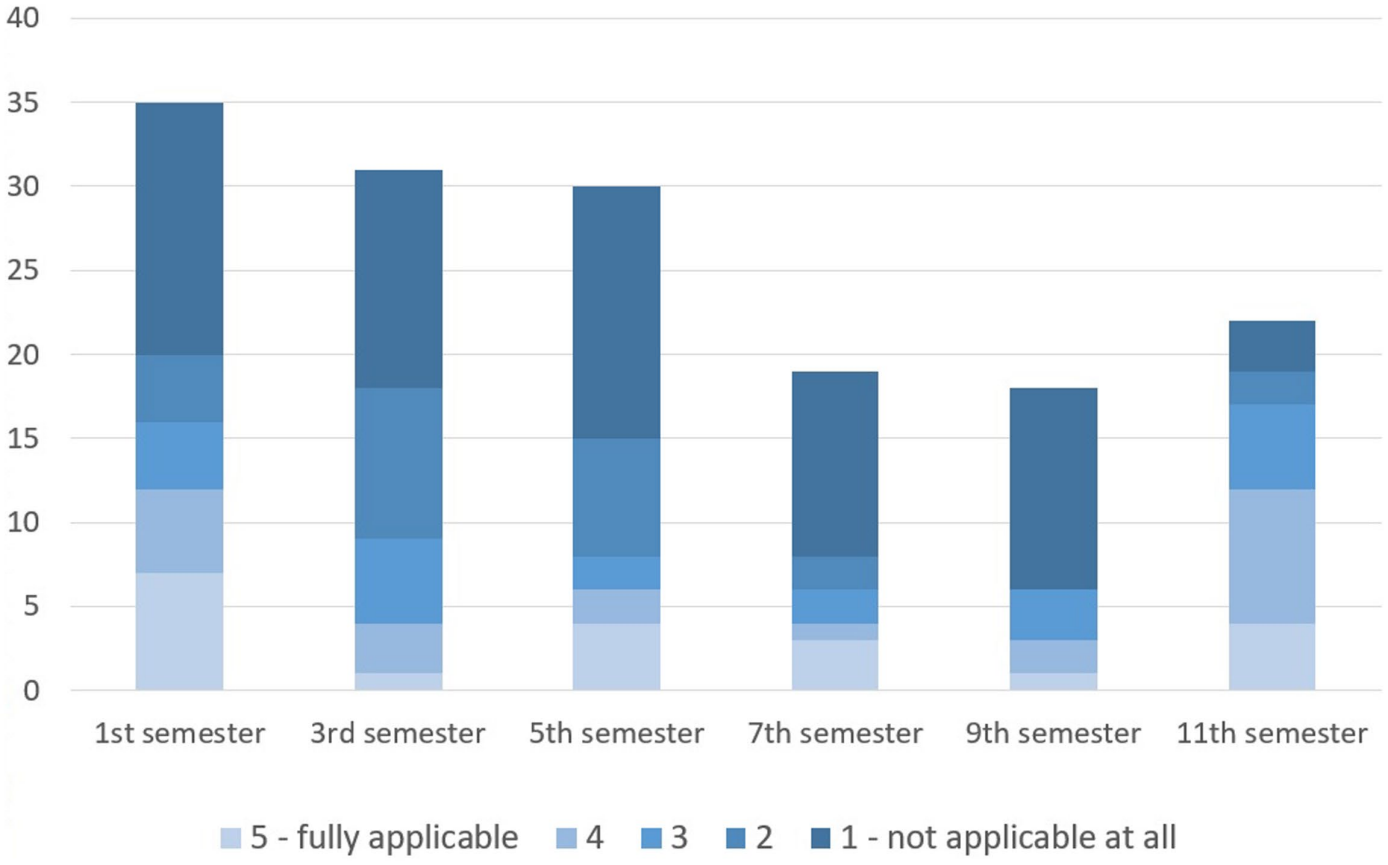
Considerations of changing the profession of those German veterinary students with thoughts of dropping out of veterinary studies (*n* = 155).

A comparison of students from different semesters shows that the proportion of students considering to discontinue their course of study increased over the first 3 years and then dropped until the end of their studies in the sixth year (Figure 7; CS: *p* = 0.030). Interestingly, students that indicated having at least occasional thoughts of discontinuing, considered to change their profession most frequently in the first and last semester (Figure 8).

#### 3.5.2 Thoughts about writing a doctoral thesis

Around a third of participants were aiming for a doctorate after their studies or had already started working on a topic (research project), while half of the students were still unsure (Table 5). It was seen that the CGS were more likely to aspire to a doctorate, while the FGS were more likely to be unsure (CS: *p* = 0.038).

**Table 5:** Responses to the question “Which of the following sentences applies best to you concerning writing a doctoral thesis or PhD?” from a survey among German veterinary students (*n* = 1,480).

	Total		CGS		FGS	
	*n*	%	*n*	%	*n*	%
I want to write a doctoral thesis after graduation	532	36.4	345	39.3	187	32.1
I already work on my doctoral thesis	36	2.5	23	2.6	13	2.2
I am not sure if I want to write a doctoral thesis	717	49.1	409	46.6	308	52.9
I do not want to write a doctoral thesis	175	12.0	101	11.5	74	12.7
Total	1,460	100	878	100	582	100

Comparison between CGS and FGS (Chi-Square test): *p* = 0.038.

## 4 Discussion

One of the main findings of this study was that 40% of veterinary students in Germany are FGS, and that FSG more frequently had financial worries when compared to CGS.

Due to voluntary participation, this survey—like most other similar studies—is potentially affected by selection bias. Even though the participants were roughly informed about the topic in the introduction to the survey, no specific hypotheses were presented there. As a result, they were unlikely to answer in line with the hypotheses subsequently addressed in the analysis. In general, almost 25% of the target population was reached. We therefore assume that although the results might not be fully representative, they provide valuable insights into German veterinary students. It should be noted that the results are not directly transferable to other countries, as admission requirements and other structures vary greatly between countries.

### 4.1 Proportions of FGS and CGS

When comparing the proportion of FGS/CGS to other countries or studies, two factors have to be considered: First, the proportion of FGS is not only dependent on the accessibility of universities for FGS but also on the proportion of parents with a college degree (25). Moreover, different definitions of first-generation students exist. We used the most common definition, defining a first-generation student as an individual whose parents did not receive a college degree (4, 14). Some other studies assess the educational background for mother and father separately (26, 27) or build more than two categories (25).

In Germany, around 72% of pupils have parents without a college degree, but only 47% of all students attending a university are FGS (28). Another study reports a proportion of 38% FGS in Germany (25). The proportion of FGS in law, medicine and pharmacology studies is significantly lower at around 32% (4). The proportion of FGS in veterinary medicine, assessed in this study (40%), can therefore been interpreted as relatively high for Germany compared to other studies with a similar degree (4).

With regard to veterinary medicine, there is a study from Australia (27) in which students at the University of Sydney and Charles Sturt University (CSU; Wagga Wagga, Australia) were compared in terms of the educational background of their parents. This study analyzed the education of mothers and fathers separately, so the data is not directly comparable. At the University of Sydney, 63% of fathers and 54% of mothers had a higher education degree, while only 38% of students at CSU had a father with such a degree, and only 24% of their mothers had a degree. The parents of students at CSU were significantly more likely to be farmers (27).

### 4.2 Admission process, school certificates & grades, and educational training

Regardless of their parents’ educational background, almost all respondents (97%) had a high school-based entrance qualification (Abitur). This is higher than the average of German students [85%; (4)]. In this study, less than 10% of respondents had first obtained a lower school-leaving qualification. This could rise to the assumption that catching up on school-leaving qualifications is not possible without complications. In order to counteract the shortage of skilled workers and help more people gain access to higher education, more information is needed on the one hand and barriers need to be removed on the other. For example, it is not always possible for adults at night schools to obtain a high school degree.

In this study, more FGSs than CGSs had completed vocational training before starting their studies. This result is consistent with other studies. Compared to children from an academically oriented home, FGS more frequently secure their educational pathway with previous vocational training (4, 22). Overall, the proportion of veterinary students who have completed vocational training is relatively high at 36%. Across Germany, 26% of students had completed vocational training before enrolling at a university for the first time (4). This means that the proportion of students who already have professional experience and therefore practical skills and knowledge is pleasingly high. At the same time, these students may have to get back into learning and need more support with learning strategies and self-organization.

Looking at school-leaving qualifications and vocational training, this study shows that more than a third of respondents have both a school-leaving qualification and another higher education entrance qualification, i.e., are dual-qualified according to the definition of the Social Survey (22). These people therefore have special knowledge and skills. It is desirable that these qualifications are given special consideration in the allocation of study places, also in order to increase diversity in veterinary medicine.

### 4.3 Financial situation, employment, and future planning

It is not only the path to studying that is paved with smaller and larger hurdles, but there are also challenges for students during their studies themselves. In this study, FGS rated their financial situation worse than CGS did with regard to various aspects. This was based on self-assessments, so it is quite possible that there are fewer differences in terms of the actual financial situation. However, generally speaking, FGS come from lower-income families (8, 10, 29). Therefore, it could also be that FGS cannot rely on the support of their parents in case of need. Another explanation is that FGS have inherently manifested financial worries mentally and are therefore more concerned about their financial situation. In general, however, the respondents tended to rate their financial situation as good—this was not the case in a survey of Australian veterinary students (30).

This study showed that around two thirds of respondents were employed alongside their studies, with no clear difference between FGS and CGS. This is roughly in line with the German average: in 2021, 64% of university students had a (part-time) job (4). This finding is surprising, as veterinary students have an above-average workload with regard to their studies and gives an insight into the demands veterinary students faced. In this study, 27% of FGS (and a lower proportion of CGS) had to finance themselves entirely through their employment, which is a considerable burden and can also have a negative impact on academic performance or mental health. Financial worries are a major cause of stress among veterinary students (31). Internationally, this proportion is difficult to compare with other studies due to differences in study-related working hours, living costs and tuition fees. In the previously cited study from Australia, 55% of veterinary students stated that they had to work alongside their studies in order to meet basic living expenses (30).

For 22% of students, short-term changes to the study schedule always or frequently lead to organizational problems. This was sometimes the case for a further 43% of respondents. Particularly in view of the high stress levels of veterinary students (32), early planning is a relief for students. One recommendation to support FGS in particular is to sensitize teachers and lecturers to the topic (14). The more that is known about the hurdles of FGS, the more likely it is that lecturers will be able to address them, e.g., by making appointments for courses and other meetings in good time and thus provide support.

In our study, 11% of students had frequent or almost constant thoughts of dropping out, which is slightly more than the average German student (4). There were no differences between FGS and CGS (Table 4). A review showed that CGS usually perform better and FGS are more likely to drop out of university (29). However, this could be distorted by other factors: In a comparison of FGS and CGS from low-income families, there was no difference in terms of academic performance (33). In this study, there were differences between FGS and CGS with regard to the reasons for dropping out: in the case of FGS, both financial reasons and the demands of studying played a role more frequently. This can be explained by the poorer assessment of their own financial situation, the feeling of “being different” (15) as well as imposter feelings of FGS (4, 9).

The percentage of students with thoughts about dropping out of studies changed only moderately over the different semesters. Interestingly, those students with thoughts of dropping out of university considered most frequently to change the profession in the first and last year of studies. Students having just completed their PY had gained insights into different fields of the veterinary profession. In Germany, students can select to some extent where they do their external practical training (EPT) during the PY, and often choose those domains (species) they are most interested in. However, some internships, like working in food safety, meat inspection or in veterinary authorities, are mandatory (34). We expected that after this phase of practical work and insights into the variety of the profession, students would have found the field where they like to work after their studies. Instead, the number of students thinking about leaving the profession increased despite the exposure to diverse fields. One reason therefore might be that students realized that their a-priori expectation differed from what they experienced during their EPT. In addition to increasing the awareness of EPT providers to communicate a realistic but positive view of the profession, it might be helpful to introduce mandatory internships before admission to veterinary school in order to better prepare students for the challenges of the profession.

This study showed that FGS were somewhat more uncertain about planning a doctorate (Table 5). This can also be explained by the reasons mentioned above, but possibly also by the older age of FGS. Not to be neglected here is the availability of knowledge about framework conditions and guidelines for doctoral studies as well as social capital (14) and financial considerations. In principle, however, significantly fewer FGS than CGS obtain a doctorate in Germany (7).

### 4.4 Conclusion

Although the topic of FGS and the hurdles they face (8–14) have been brought into focus in recent years, even today not everyone is aware of this topic. This is illustrated by the fact that this study is one of the first studies ever dealing with FGS in veterinary medicine. It is important to create an awareness of social background at universities in order to counteract challenges for FGS and to support them when family structures or friends can no longer help. For this reason, the first step is to create more awareness and understanding of this topic among lecturers, but also among the students themselves. One way to support FGS is to establish mentoring programs, as in the USA (35, 36). In Germany, there are already mentoring programs (11) for FGS, but not directly in veterinary medicine. Further help could be provided by courses and financial counselling, as well as more forward planning for appointments and courses. It should be borne in mind that FGS also experience disadvantages after they finished their studies: FGS earn less even with the same educational qualifications [class-pay-gap; (37)]. The extent to which this also applies to veterinarians is unclear and should be investigated in further studies. It could be useful to support FGS both before their studies and at the beginning of their career. Although it has been found that FGS are more likely to have completed a vocational training prior to studying, there is a lack of research into the extent to which this additional qualification impacts on the labor market and improves diversity through prior practical experience. However, this also requires a better overview, for example by recording educational background during enrolment. This study has closed a major milestone in the knowledge gap. In summary, it can be said that more attention needs to be paid to this topic in order to bring about further improvements, particularly for the future of the veterinary labor market.

## Data Availability

The datasets presented in this study can be found in online repositories. The names of the repository/repositories and accession number(s) can be found at: https://box.fu-berlin.de/s/bzpMoTHGSMaYLAX.

## Footnotes

^1^ https://box.fu-berlin.de/s/bzpMoTHGSMaYLAX
