# Supplementary material for: Disparities in veterinary education: a survey comparing first-generation and continuing-generation students in Germany
Source: Front Vet Sci. 2025 Jun 25;12:1595643. doi: 10.3389/fvets.2025.1595643 (PMC12238717; doi:10.3389/fvets.2025.1595643)
Supplement: Supplementary file 1 [file Data_Sheet_1.pdf]

LimeSurvey

Herzlich willkommen!

Vielen Dank, dass Sie an meiner, gemeinsam mit dem Institut für Veterinär-Epidemiologie & Biometrie der Freien Universität Berlin, durchgeführten freiwilligen Umfrage bei Studierenden der fünf deutschen Veterinärmedizin-Bildungsstätten teilnehmen möchten.

Wir erleben einen zunehmenden Mangel an Tierärztinnen und Tierärzten im Berufsstand bei gleichbleibenden Zulassungszahlen für das Studium, aber leicht zurückgehenden Absolventenzahlen. Gleichzeitig wurden in den letzten Jahren die Zulassungskriterien (Abiturientenquote, Einbeziehung des TMS in das Auswahlverfahren der Hochschulen, Berufsqualifikation etc.) angepasst.

Im aktuellen 11., 9. und 7. Semester befinden sich überwiegend Studierende, welche noch nicht am TMS (Medizinertest; Test für medizinische Studiengänge) teilgenommen haben, während in niedrigeren Semestern die meisten Studierenden den TMS absolviert haben.

Vor diesem Hintergrund möchten wir mittels einer repräsentativen Umfrage unter den Veterinärmedizin-Studierenden der fünf Vet.-Med.-Bildungsstätten in Deutschland erfassen, auf welchem Weg diese Studierenden einen Studienplatz erhalten haben, welchen Bildungshintergrund sie jeweils hatten, und ob sich die Kohorten über Zeit oder bezüglich des Zulassungsweges in den im Studium wahrgenommenen Herausforderungen unterscheiden. In dem 15-minütigen Fragebogen erwarten Sie neben Fragen bspw. zu Ihrem Schulabschluss und dem Bildungsstand Ihrer Eltern auch solche zu den aktuellen Lebens- und Studienumständen, sowie besonderen Belastungen beispielsweise durch Studien- und Praktikumsorganisation. Basierend darauf soll auch die Situation von sogenannten First-Generation-Students (FSG; “Arbeiterkinder“) im Studiengang Veterinärmedizin genauer beleuchtet werden.

Verglichen werden die Resultate unserer Studie mit relevanten Teilergebnissen der deutschlandweiten 21. Sozialerhebung und der ganz neuen „Eine für alle“ (efa) - Erhebung, um mögliche Unterschiede von Studierenden der Tiermedizin zu Studierenden anderer Fächer darzustellen. Der Fokus liegt hierbei neben den Auswirkungen des TMS auf Studieneinstieg und Studienerfolg auch auf den sozialen Hintergründen der Studierenden, insbesondere der Unterschiede von Studierenden aus sogenannten Akademiker-Familien im Vergleich zu FGS.

Alle vollständig ausgefüllten Fragebögen fließen in die Bewertung ein und erhöhen die Aussagekraft der Studie. Machen Sie daher mit, denn jede Stimme zählt!

Vielen Dank!

Teil A: A. Demografische Daten

Allgemeine Fragen zu Ihrer Person.

A1. In welchem Jahr wurden Sie geboren?

Bsp. 1987

☐☐☐☐

A2. Welchem Geschlecht fühlen Sie sich zugehörig?

- Weiblich ☐
- Männlich ☐
- ich möchte/ kann mich keinem der beiden Geschlechter zuordnen ☐

A3. Welchen Familienstand haben Sie?

- ledig ☐
- verheiratet/ eingetragene Lebenspartnerschaft ☐
- geschieden ☐
- verwitwet ☐
- keine Angabe ☐

A4. Sind Sie für die Betreuung von minderjährigen Kindern in Ihrem privaten Umfeld (mit) verantwortlich?

- Ja ☐
- Nein ☐

A5. Je nach Alter und Lebenssituation ist die finanzielle Situation der Studierenden sehr unterschiedlich.

Wie bewerten Sie Ihre derzeitige finanzielle Situation?

- 1 - sehr gut; problemlos ☐
- 2 ☐
- 3 ☐
- 4 ☐
- 5 - schlecht; stark belastend ☐
- keine Angabe ☐

LimeSurvey

A6. **Waren Sie in den letzten 12 Monaten neben dem Studium erwerbstätig?**

- Nein ☐
- Ja; regelmäßig, aber nur in der vorlesungsfreien Zeit ☐
- Ja, regelmäßig sowohl während des Semesters, als auch in der vorlesungsfreien Zeit ☐
- gelegentlich; bei Bedarf ☐

A7. **In welchem Umfang waren Sie in den letzten 12 Monaten erwerbstätig?**

- Mini-Job ☐
- Teilzeit/ Werkstudent (60 Std im Monat) ☐
- Teilzeit/ Werkstudent (80 Std im Monat) ☐
- Teilzeit/ Werkstudent (100 Std im Monat; oder mehr) ☐
- Vollzeit (40 Std Woche; 160 Std im Monat) ☐
- Sonstiges: ☐

Sonstiges:

A8. **Welche Gründe haben Sie für die Erwerbstätigkeit?**

- Ich bin auf eine Erwerbstätigkeit angewiesen, um mein Studium zu finanzieren (alleinige Einnahme) ☐
- BAFöG/ Stipendium/ Kredit alleine reicht nicht aus ☐
- Meine Eltern können die Kosten für mein Studium nicht/ nicht ganz tragen ☐
- Durch die Erwerbstätigkeit kann ich mir zusätzliches leisten (z.B. Auto, Urlaub etc.) ☐
- Um praktische Erfahrungen zu sammeln/ Kontakte für eine spätere Beschäftigung zu knüpfen ☐
- Sonstiges: ☐

Sonstiges:

A9. **Welchen Einfluss hat die Erwerbstätigkeit auf Ihr Studium?**

	1 - trifft nicht zu	2	3	4	5 - trifft voll und ganz zu
Arbeiten zu gehen ist ein guter Ausgleich zum Studium	☐	☐	☐	☐	☐

	1 - trifft nicht zu	2	3	4	5 - trifft voll und ganz zu
Arbeiten zu gehen belastet mich, weil dadurch Zeit zum Lernen und Ausruhen fehlt	☐	☐	☐	☐	☐

A10. Über wen sind Sie aktuell krankenversichert?

- Eltern ☐
- Partner ☐
- Arbeitgeber ☐
- Gesetzlich versichert ☐
- Privat versichert ☐
- Keine Angabe ☐
- Sonstiges ☐

Sonstiges

[ ]

A11. Welchen Einfluss haben die zusätzlichen Kosten der Krankenversicherung für Sie?

- 1 - es macht mir nichts aus ☐
- 2 ☐
- 3 ☐
- 4 ☐
- 5 - es belastet mich sehr ☐

Teil B: B. Fragen zur Studienwahl, Zulassung und aktuellem Studienstatus

Fragen zur Studienbewerbung und Voraussetzungen

B1. Nennen Sie bitte Ihren aktuellen Studienort.

- Berlin, Freie Universität ☐
- Gießen, Justus- Liebig- Universität ☐
- Hannover, Stiftung Tierärztliche Hochschule ☐
- Leipzig, Universität ☐
- München, Ludwig- Maximilians- Universität ☐

LimeSurvey

B2. **Wann haben Sie angefangen Veterinärmedizin zu studieren?**

- vor 2015 ☐
- 2015 ☐
- 2016 ☐
- 2017 ☐
- 2018 ☐
- 2019 ☐
- 2020 ☐
- 2021 ☐
- 2022 ☐

B3. **Nennen Sie bitte Ihr aktuelles Fachsemester.**

- 1.Semester ☐
- 3. Semester ☐
- 5. Semester ☐
- 7. Semester ☐
- 9. Semester ☐
- 11.Semester ☐

B4. **Welchen höchsten Schulabschluss hatten Sie vor Beginn des Studiums erworben?**

Bitte kreuzen Sie Ihren Abschluss an, auch wenn Sie über die Berufserfahrung oder andere Auswahlverfahren Ihren Studienplatz bekommen haben.

- Allgemeine Hochschulreife ☐
- fachgebundene Hochschulreife ☐
- Fachhochschulreife ☐
- Mittlere Reife ☐
- Hauptschulabschluss ☐
- kein Abschluss ☐
- anderer Abschluss ☐

B5. **Nennen Sie bitte den Zeitpunkt des Erwerbs, sowie die Durchschnittsnote Ihrer allgemeinen Hochschulreife.**

Punktzahl bitte in Note umrechnen.

Jahr (z.B. 2020) ☐☐☐☐☐☐☐☐☐☐

Durchschnittsnote (z.B. 2,0) ☐☐☐☐☐☐☐☐☐☐

LimeSurvey

B6. Haben Sie vor dem in Frage 15 genannten höchsten Schulabschluss bereits einen niedrigeren Abschluss erworben?

(Bitte kreuzen Sie den niedrigsten zuvor erworbenen Abschluss an, auch wenn Sie noch weitere erworben haben) z.B. Sie haben die Gesamtschule mit einem Realschulabschluss abgeschlossen und in späteren Jahren Ihr Abitur an einem Abendgymnasium nachgeholt.

- [ ] Nein, ich habe keinen niedrigeren Abschluss erworben
- [ ] fachgebundene Hochschulreife
- [ ] Fachhochschulreife
- [ ] Mittlere Reife (ausgenommen Gymnasiasten, welche automatisch vor dem Übergang zur Oberstufe einen Mittlere Reife Abschluss erlangt haben)
- [ ] Hauptschulabschluss
- [ ] Ich habe die Schule ohne Abschluss verlassen
- [ ] anderer Abschluss

B7. Welche Gründe gab es für den niedrigeren Abschluss?

- [ ] Vorher andere Ziele (z.B. Ausbildung, wofür man kein Abitur benötigt)
- [ ] Keine Lust zu lernen/ Ich wusste nicht, wofür ich mich anstrengen sollte
- [ ] Familienprobleme/ Beziehungsprobleme
- [ ] Krankheit/ Schwangerschaft
- [ ] Mobbing
- [ ] keine Angabe
- [ ] Sonstige

Sonstige

____

B8. Haben Sie vor dem Veterinärmedizinstudium bereits eine oder mehrere Berufsausbildung/en abgeschlossen?

- [ ] Nein
- [ ] Ja, eine Ausbildung
- [ ] Ja, zwei Ausbildungen
- [ ] Ja, drei Ausbildungen

B9. Warum haben Sie eine Berufsausbildung angefangen?

- [ ] Allgemeine/ fachgebundene Hochschulreife nicht ausreichend für die Zulassung um Veterinärmedizin zu studieren
- [ ] Wollte erst Berufserfahrung sammeln
- [ ] Wusste nicht, was ich machen sollte

Ich brauchte die Ausbildung, um am Abendgymnasium das Abitur nachzuholen ☐

Haben mir Eltern/Freunde geraten ☐

Vor dem Studium andere Ziele ☐

Sonstiges ☐

B10. Wählen Sie bitte Ihren abgeschlossenen Ausbildungsberuf.

Für Alle mit mehreren Ausbildungsberufen: wählen Sie bitte Ihren ersten Ausbildungsberuf.

Der Einfachheitshalber haben wir sämtliche im Veterinärmedizinstudium anerkannten Ausbildungen ausführlich aufgelistet. Andere Ausbildungen zählen zu: "nicht im Veterinärmedizinstudium anerkannten Ausbildung", also z.B. Köchin.

Nicht im Veterinärmedizinstudium anerkannte Ausbildung ☐

Anästhesietechnische/r Assistent/in ☐

Biologielaborant/in ☐

Chemielaborant/in ☐

Fischwirt/in ☐

Fleischer/in ☐

Landwirt/in ☐

Medizinisch-technische/r Assistent/in -Funktionsdiagnostik ☐

Medizinisch-technische/r Assistent/in (MTA) ☐

Medizinisch-technische/r Laboratoriumsassistent/in ☐

Medizinisch-technische/r Radiologieassistent/in ☐

Medizinlaborant/in ☐

Operationstechnische/r Angestellte/r ☐

Operationstechnische/r Assistent/in ☐

Pferdewirt/in ☐

Tierarzthelfer/in ☐

Tiermedizinische/r Fachangestellte/r ☐

Tierpfleger/in ☐

Tierwirt/in ☐

Veterinärmedizinisch-technische/r Assistent/in ☐

LimeSurvey

B11. Bitte nennen Sie Ihren zweiten abgeschlossenen Ausbildungsberuf.

- Nicht im Veterinärmedizinstudium anerkannte Ausbildung ☐
- Anästhesietechnische/r Assistent/in ☐
- Biologielaborant/in ☐
- Chemielaborant/in ☐
- Fischwirt/in ☐
- Fleischer/in ☐
- Landwirt/in ☐
- Medizinisch-technische/r Assistent/in -Funktionsdiagnostik ☐
- Medizinisch-technische/r Assistent/in (MTA) ☐
- Medizinisch-technische/r Laboratoriumsassistent/in ☐
- Medizinisch-technische/r Radiologieassistent/in ☐
- Medizinlaborant/in ☐
- Operationstechnische/r Angestellte/r ☐
- Operationstechnische/r Assistent/in ☐
- Pferdewirt/in ☐
- Tierarzthelfer/in ☐
- Tiermedizinische/r Fachangestellte/r ☐
- Tierpfleger/in ☐
- Tierwirt/in ☐
- Veterinärmedizinisch-technische/r Assistent/in ☐

LimeSurvey

B12. Bitte nennen Sie Ihre dritte abgeschlossene Ausbildung.

- Nicht im Veterinärmedizinstudium anerkannte Ausbildung ☐
- Anästhesietechnische/r Assistent/in ☐
- Biologielaborant/in ☐
- Chemielaborant/in ☐
- Fischwirt/in ☐
- Fleischer/in ☐
- Landwirt/in ☐
- Medizinisch-technische/r Assistent/in -Funktionsdiagnostik ☐
- Medizinisch-technische/r Assistent/in (MTA) ☐
- Medizinisch-technische/r Laboratoriumsassistent/in ☐
- Medizinisch-technische/r Radiologieassistent/in ☐
- Medizinlaborant/in ☐
- Operationstechnische/r Angestellte/r ☐
- Operationstechnische/r Assistent/in ☐
- Pferdewirt/in ☐
- Tierarzthelfer/in ☐
- Tiermedizinische/r Fachangestellte/r ☐
- Tierpfleger/in ☐
- Tierwirt/in ☐
- Veterinärmedizinisch-technische/r Assistent/in ☐

B13. Haben Sie vor Beginn des Veterinärmedizinstudiums eine Berufsausbildung begonnen und ohne Abschluss beendet?

- Nein ☐
- Ja, eine Ausbildung ☐
- Ja, zwei Ausbildungen ☐
- Ja, drei Ausbildungen ☐

LimeSurvey

B14. **Welchen Ausbildungsberuf haben Sie vor Beginn des Veterinärmedizinstudiums angefangen?**

Wenn Sie mehr als eine abgebrochene Ausbildung haben, nennen Sie bitte Ihren ersten Ausbildungsberuf.

B15. **Bitte nennen Sie den Ausbildungsberuf Ihrer zweiten abgebrochenen Ausbildung.**

B16. **Bitte nennen Sie den Ausbildungsberuf Ihrer dritten abgebrochenen Ausbildung.**

B17. **Haben Sie vor Beginn des Veterinärmedizinstudiums schon mindestens ein anderes Studium angefangen und entweder abgebrochen oder abgeschlossen?**

Nein ☐

Ja ☐

B18. **Bitte nennen Sie das jeweilige Studienfach und fügen Sie hinzu, ob Sie dieses abgeschlossen oder abgebrochen haben.**

z.B. Zahnmedizin nach 4 Semestern abgebrochen.

B19. Nennen Sie bitte das Verfahren der Studienplatzvergabe, über welches Sie die Zulassung zum Tiermedizinstudium erhalten haben.

- Abiturbestenquote ☐
- Auswahlverfahren der Hochschule (AdH) (Note der Hochschulzugangsberechtigung; TMS -ab 2020; Abgeschlossene Ausbildung; Berufstätigkeit) ☐
- Zusätzliche Eignungsquote (ZEQ) (TMS -ab 2020; Abgeschlossene Ausbildung; Berufstätigkeit; Wartezeit; Preise in Wettbewerben) ☐
- Ausländische Bewerber Quote (kein EU- Staat) ☐
- Härtefall ☐
- Zweitstudium ☐
- Losverfahren ☐
- Keine Angabe ☐

B20. Haben Sie am TMS (Medizinertest) teilgenommen?

- Ja ☐
- Nein ☐

B21. Wie oft haben Sie am TMS teilgenommen?

- Ein Mal ☐
- Zwei Mal ☐
- Keine Angabe ☐

B22. Nennen Sie bitte das Jahr, in welchem Sie am TMS teilgenommen haben, sowie den jeweiligen erreichten Prozentwert. 1. Mal

Jahr ☐☐☐☐☐☐☐☐☐☐

Prozentwert ☐☐☐☐☐☐☐☐☐☐

B23. Nennen Sie bitte das Jahr, in welchem Sie am TMS teilgenommen haben, sowie den jeweiligen erreichten Prozentwert. 2. Mal

Jahr ☐☐☐☐☐☐☐☐☐☐

Prozentwert ☐☐☐☐☐☐☐☐☐☐

B24. Welche Vorbereitungen haben Sie für den Medizinertest getroffen?

- Flyer ☐
- Bezahlte Kurse ☐
- Selbststudium ☐
- Bücher käuflich erworben (auch, wenn Angehörige dies bezahlt haben) ☐
- Bücher geschenkt bekommen ☐
- Kostenlose Kurse (z.B. Onlinevideos) ☐

Private bezahlte Nachhilfe ☐

Private kostenlose Nachhilfe (z.B. Eltern, Freunde; Klassenkameraden) ☐

Sonstiges ☐

Keine ☐

B25. Warum haben Sie am TMS teilgenommen?

Ich brauchte die Punkte, um meinen NC aufzubessern ☐

Ich brauchte die Punkte, weil ich über die Berufserfahrung sonst zu wenige hatte ☐

Ich brauchte die Punkte aus anderen Gründen ☐

Weil das Verfahren neu war und ich nicht wusste, welchen Einfluss der TMS auf die Platzvergabe hat ☐

Ich habe aus Sicherheit mitgemacht, weil sonst andere mit einem schlechteren NC trotzdem bessere Chancen haben könnten ☐

Meine Familie/ Freunde/ Partnerin haben mir dazu geraten ☐

Weil Freunde/ Klassenkameraden etc. am TMS teilgenommen haben ☐

Sonstiges: ☐

Sonstiges:

B26. Wie empfandest du den Mediziniertest insgesamt?

leicht ☐

machbar ☐

schwer ☐

keine Angabe ☐

B27. Warum haben Sie nicht am TMS teilgenommen?

Unnötig, weil Abitur Bestnote ☐

Unnötig, weil Härtefallantrag ☐

Unnötig, weil Zweitstudium ☐

Unnötig, weil Ausländerquote ☐

Kein Geld für den Test selbst ☐

Kein Geld für Vorbereitungskurse ☐

Keine Zeit zum Vorbereiten ☐

Keine Zeit für den Test ☐

Kein Interesse ☐

Ich wusste nicht/ zu spät von dem TMS ☐

Sonstiges: ☐

Sonstiges:

B28. Seit dem Wintersemester 2020/21 wurde der TMS (Medizinertest) auch für Tiermediziner in Deutschland etabliert; bitte bewerten Sie die folgenden Aussagen zum Medizinertest.

	1 - finde ich schlecht	2	3	4	5 - finde ich gut
Ein TMS- Ergebnis ist kein einheitlicher Maßstab, weil er lediglich zwischen allen Testpersonen vom selben Tag vergleicht (werden am Folgetag schlechtere Testpersonen getestet, könnte der eigene Schnitt an dem Tag besser ausfallen)	☐	☐	☐	☐	☐
Der TMS stellt die Studierfähigkeit dar (z.B. Leistungsfähigkeit unter Zeitdruck)	☐	☐	☐	☐	☐
Der Test ist nicht spezifisch auf Tiermedizin ausgerichtet	☐	☐	☐	☐	☐
Man hat zwei Mal die Chance, den TMS zu absolvieren	☐	☐	☐	☐	☐
Der TMS kompensiert einen schlechteren NC	☐	☐	☐	☐	☐
Man braucht kein Fachwissen	☐	☐	☐	☐	☐
Man kann für den Test üben	☐	☐	☐	☐	☐

B29. Inwieweit stimmen Sie folgenden Aussagen zum Medizinertest zu?

	1 - stimme nicht zu	2	3	4	5 - stimme voll und ganz zu
Der Medizinertest spiegelt lediglich wider, wie gut sich die Personen etwas merken können oder etwas theoretisch anwenden können, jedoch fehlt vollkommen die praktische Anwendung, was für das Veterinärmedizinstudium wichtig ist	☐	☐	☐	☐	☐
Der TMS kostet Geld (Test selbst und gute Vorbereitungskurse)	☐	☐	☐	☐	☐
Es ist ein erheblicher Zeitaufwand neben/ nach dem Abitur	☐	☐	☐	☐	☐

B30. Studierende, welche vor dem Studium eine Ausbildung absolvierten, können im Studium erworbenes Wissen unter Umständen schneller oder besser praktisch anwenden. Um diese Zielgruppe zu fördern und dem theoretischen Medizinertest einen stärker gewichteten Gegenpart aufzustellen, wäre eine Option, Ausbildungen mehr Punkte zuzuschreiben.

Inwieweit stimmen Sie einern stärkere Bewertung einer für das Tiermedizinstudium anerkannten Ausbildung bzw. generell einer Ausbildung bei der Bewertung der Punkte für das Auswahlverfahren der Hochschule (AdH) zu?

	1 - stimme nicht zu	2	3	4	5 - stimme voll und ganz zu
Alle Ausbildungen sollten mit mehr Punkten bewertet werden (z.B. auch Köchin etc.)	☐	☐	☐	☐	☐
Nur die tiermedizinisch anerkannten Ausbildungen sollten mit mehr Punkten bewertet werden	☐	☐	☐	☐	☐

B31. Wenige Studierende haben Erfahrungen im Berufsfeld der Tiermedizin. Dies kann durch falsche Erwartungen zu einer Desillusionierung im oder nach dem Studium führen. Um dem entgegenzuwirken, könnten nachgewiesene Praktika im tiermedizinischen Bereich (z.B. ab dem ersten Schulabschluss – Hauptschulabschluss/ Realschulabschluss/ Abitur) zusätzliche Punkte für die Studienbewerbung ergeben, die neben dem TMS (Studierfähigkeitstest) auch zeigen, dass Studierende sich mit der praktischen Tätigkeit des Berufsfeldes auseinandergesetzt haben.

Wie würden Sie die Etablierung zusätzlicher Punkte für praktische Erfahrung für die Studienbewerbung bewerten?

- ☐ 1 - finde ich schlecht
- ☐ 2
- ☐ 3
- ☐ 4
- ☐ 5 - finde ich gut

LimeSurvey

B32. Haben Sie innerhalb der letzten 12 Monate nach einem Sonderstudienplan studiert oder ein reduziertes Studium in der Tiermedizin absolviert?

- Kein reduziertes Pensum (Verlauf gemäß Studienordnung) ☐
- Ein- oder mehrere Urlaubssemester ☐
- Sonderstudienplan ☐
- Sonstiges: ☐

Sonstiges:

Teil C: C. Studienbezogene Fragen während des Studiums

Diese Fragengruppe befasst sich inhaltlich mit einer allgemeinen Einschätzung des Studiums insgesamt sowie der Thematik des Aufbaus des Studiums.

C1. Welche der folgenden finanziellen Aspekte bereitete Ihnen in den letzten 12 Monaten im Studium Sorgen?

	1 - trifft nicht zu	2	3	4	5 - trifft voll und ganz zu
Semesterbeiträge	☐	☐	☐	☐	☐
Finanzielle Überbrückung des praktischen Jahres (PJ)	☐	☐	☐	☐	☐
Wegfall der Erwerbstätigkeit	☐	☐	☐	☐	☐
Wohnung	☐	☐	☐	☐	☐
Familie/ Kinder	☐	☐	☐	☐	☐
Mobilität	☐	☐	☐	☐	☐

C2. Welche der folgenden organisatorischen Aspekte bereitete Ihnen in den letzten 12 Monaten im Studium Sorgen?

	1 - trifft nicht zu	2	3	4	5 - trifft voll und ganz zu
Aufbau/ Ablauf des Studiums	☐	☐	☐	☐	☐
Wohnungssuche	☐	☐	☐	☐	☐
Bedenken bezüglich der Planung im PJ	☐	☐	☐	☐	☐

	1 - trifft nicht zu	2	3	4	5 - trifft voll und ganz zu
Alltagsorganisation und Eigenverantwortung	☐	☐	☐	☐	☐

C3. Inwieweit bereiten Ihnen folgende Aspekte bezüglich des PJ Sorgen; bzw. Inwieweit haben Ihnen folgende Aspekte vor Ihrem PJ Sorgen bereitet?

	1 - trifft nicht zu	2	3	4	5 - trifft voll und ganz zu
Keine Zeit, nebenher für die Staatsexamensprüfungen zu lernen	☐	☐	☐	☐	☐
Unsicherheit, in welche Richtung man Praktika wählen möchte	☐	☐	☐	☐	☐
Finanzielle Überbrückung ohne Erwerbstätigkeit	☐	☐	☐	☐	☐
Verlust der Erwerbstätigkeit (wenn Arbeitgeber längeres Aussetzen nicht tragen kann)	☐	☐	☐	☐	☐
Doppelbelastung bei z.B. Unterkunft für das Praktikum und WG am Studienort	☐	☐	☐	☐	☐
Entfernung mancher Praktika-Plätze (lange Fahrtzeit und damit verbundene Kosten)	☐	☐	☐	☐	☐
Eigenverantwortliche Planung des PJ	☐	☐	☐	☐	☐
Unterbringung des Kindes/ der Kinder	☐	☐	☐	☐	☐
Versorgung des Tieres/ der Tiere	☐	☐	☐	☐	☐
Pflegeverantwortung für Angehörige	☐	☐	☐	☐	☐

C4. Studierende, welche neben dem Studium erwerbstätig sein müssen, sich um eine Kinderbetreuung kümmern müssen oder anderweitige Verpflichtungen haben, können durch eine zu späte Kurseinteilung, Planänderungen oder kurzfristige Terminvergabe von Prüfungsvorbereitungsgesprächen organisatorische Probleme bekommen. Inwieweit betrifft Sie dieses Problem?

Kurzfristige Termine, Kurseinteilungen oder Planänderungen:

- bereitet mir nie Probleme ☐
- bereitet mir manchmal Probleme ☐
- bereitet mir oft Probleme ☐
- bereitet mir immer Probleme ☐
- Eine zu späte Kurseinteilung, Planänderungen oder kurzfristige Terminvergabe von Prüfungsvorbereitungsgesprächen habe ich nie erlebt ☐

C5. Welche Probleme haben Sie bei einer kurzfristigen Kurseinteilung, Planänderung oder kurzfristigen Terminvergabe von Prüfungsvorbereitungsgesprächen?

Ich habe Probleme/ Schwierigkeiten bei:

	1 - trifft nicht zu	2	3	4	5 - trifft voll und ganz zu
Der Unterbringung meines Kindes	☐	☐	☐	☐	☐
Der Umänderung der Schicht bei der Arbeit	☐	☐	☐	☐	☐
Der Pflege und Versorgung von Angehörigen	☐	☐	☐	☐	☐
Organisation von Mitfahrgelegenheiten	☐	☐	☐	☐	☐
Arzttermine / Behördengänge	☐	☐	☐	☐	☐
Andere Termine wie z.B. Wohnungsbesichtigung, Bewerbungsgespräche, Handwerkertermine etc.	☐	☐	☐	☐	☐
Private Termine (z.B. Familientreffen, Trauerfeier, Vereinstreffen, Hilfsorganisationsveranstaltungen etc.)	☐	☐	☐	☐	☐

C6. Haben Sie in den letzten 12 Monaten darüber nachgedacht, das Veterinärstudium abzubrechen?

1 - überhaupt nicht ☐

2 - gelegentlich ☐

3 - häufiger ☐

4 - praktisch durchgehend ☐

C7. Wie wichtig sind die folgenden Gründe für Ihre Überlegung, Ihr aktuelles Studium abzubrechen?

	1 - trifft nicht zu	2	3	4	5 - trifft voll und ganz zu
finanzielle Probleme	☐	☐	☐	☐	☐
zu hohe Studienanforderungen	☐	☐	☐	☐	☐
Veränderung meiner beruflichen Orientierung	☐	☐	☐	☐	☐
Studiengangwechsel	☐	☐	☐	☐	☐
Schwangerschaft- Kindererziehung	☐	☐	☐	☐	☐
Betreuung pflegebedürftiger Angehöriger	☐	☐	☐	☐	☐
akute gesundheitliche Probleme	☐	☐	☐	☐	☐
chronische Krankheit/ Behinderung	☐	☐	☐	☐	☐

C8. **Welche der folgenden Aussagen bezüglich Promotion (Dr. med. vet. oder PhD) trifft auf Sie derzeit am besten zu?**

- Ich strebe nach dem Studium eine Promotion an. ☐
- Ich arbeite derzeit bereits an einem Dissertationsthema. ☐
- Ich überlege noch, ob eine Promotion für mich infrage kommt. ☐
- Ich möchte nicht promovieren. ☐
- Keine Angabe ☐
- Sonstiges: ☐

Sonstiges:

Teil D: D. Sozialer Hintergrund – Familiäre Einordnung

D1. **Welches ist der höchste allgemeinbildende Schulabschluss Ihrer Mutter/ Ihres Vaters?**

(Quelle: in Anlehnung an die 21. Sozialerhebung, sowie der "efa")

	Mutter	Vater
allgemeine Hochschulreife (Abitur)	☐	☐
Fachhochschulreife	☐	☐
Realschulabschluss, mittlere Reife, 10. Klasse polytechnische Oberschule	☐	☐
Haupt-, Volksschulabschluss (min. 8. Klasse)	☐	☐
anderer Schulabschluss	☐	☐
hat keinen Schulabschluss	☐	☐
weiß ich nicht	☐	☐

D2. **Haben Ihre Eltern vorher einen niedrigeren Abschluss gehabt?**

z.B. vorher einen Realschulabschluss, dann das Abitur nachgeholt

	Mutter	Vater
Ja	☐	☐
Nein	☐	☐
weiß ich nicht	☐	☐

D3. **Welches ist der höchste akademische oder berufliche Abschluss Ihres Vaters/ Ihrer Mutter?**

(Quelle: in Anlehnung an die 21. Sozialerhebung, sowie der "efa")

	Mutter	Vater
Promotion (Doktortitel)	☐	☐
Abschluss an einer Universität (Diplom; Bsc., Msc.)	☐	☐
Fachhochschulabschluss	☐	☐
Abschluss an einer Fach-, Meister-, Technikerschule, Berufs-, Fachakademie, Schule des Gesundheitswesens	☐	☐
Lehre bzw. Facharbeiterabschluss	☐	☐
anderer beruflicher Abschluss	☐	☐
hat keine Berufsausbildung	☐	☐
Darüber habe ich keine Kenntnis	☐	☐

Teil E: E. Persönlichkeit

Diese Fragengruppe befasst sich mit Ihrer Persönlichkeit.

E1. **Inwieweit treffen die folgenden Aussagen auf Sie zu?**

(Quelle: in Anlehnung an die 21. Sozialerhebung, sowie der "efa")

	1 - trifft nicht zu	2	3	4	5 - trifft voll und ganz zu
Ich bin bequem, neige zur Faulheit	☐	☐	☐	☐	☐
Ich werde leicht nervös und unsicher	☐	☐	☐	☐	☐
ich gehe aus mir raus, bin gesellig	☐	☐	☐	☐	☐
ich erledige Aufgaben gründlich	☐	☐	☐	☐	☐
ich bin eher zurückhaltend, reserviert	☐	☐	☐	☐	☐

E2. **Inwieweit treffen die folgenden Aussagen auf Sie zu?**

(Quelle: in Anlehnung an die 21. Sozialerhebung, sowie der "efa")

	1 - trifft nicht zu	2	3	4	5 - trifft voll und ganz zu
In schwierigen Situationen kann ich mich auf meine Fähigkeiten verlassen.	☐	☐	☐	☐	☐
Die meisten Probleme kann ich aus eigener Kraft gut meistern.	☐	☐	☐	☐	☐
Auch anstrengende und komplizierte Aufgaben kann ich in der Regel gut lösen.	☐	☐	☐	☐	☐

LimeSurvey

E3. Wenn Sie sich selbst beschreiben müssten: Inwiefern treffen die folgenden Eigenschaften auf Sie zu?

(Quelle: in Anlehnung an die 21. Sozialerhebung, sowie der "efa")

	1 - trifft nicht zu	2	3	4	5 - trifft voll und ganz zu
selbstbewusst	☐	☐	☐	☐	☐
einfühlsam	☐	☐	☐	☐	☐
resilient	☐	☐	☐	☐	☐

E4. Herzlichen Dank für Ihre Antworten.Haben Sie noch Fragen oder Anregungen zur Umfrage?

Herzlichen Dank für die Teilnahme und viel Erfolg im Studium!
